# Fertility counselling and fertility preservation among early onset female cancer patients—A Finnish register‐based study

**DOI:** 10.1002/cam4.7034

**Published:** 2024-02-24

**Authors:** Johanna Melin, Elina Holopainen, Hanna Savolainen‐Peltonen

**Affiliations:** ^1^ Department of Obstetrics and Gynecology University of Helsinki, Helsinki University Hospital Helsinki Finland; ^2^ Monash Centre for Health Research and Implementation School of public Health and Preventive Medicine Monash University Melbourne Victoria Australia

**Keywords:** controlled ovarian hyperstimulation, embryo cryopreservation, female cancer patients, fertility counselling, fertility preservation, oocyte cryopreservation

## Abstract

**Introduction:**

Advances in multimodality cancer treatments have increased long‐term survival rates for early onset cancer patients, with 5‐year survival rates reaching 80% in Northern Europe. According to recent recommendations, clinicians should, as early as possible, inform cancer patients about the impact that cancer treatment may have on their fertility. Still, there is limited published data on fertility counselling (FC) and fertility preservation (FP) for cancer patients.

**Methods:**

This register‐based study used hospital records to identify female cancer patients in the hospital district (*n* = 192) who received FC at the age of 16–42 years between 2011 and 2019.

**Results:**

Altogether, 97 (50.5%) cancer patients were eligible for FP. Of these, 55 (56.7%) underwent FP, whereas 42 (43.3%) declined. Women undergoing FP were recommended cancer treatments with a higher risk of infertility (*p* = 0.01), and women with breast cancer were more prone to undergo FP than women with lymphoma (*p* = 0.043). In FP treatment cycles, the mean number of oocytes retrieved (13.9 ± 7.7 vs. 12.0 ± 6.5, *p* = 0.04) and transferrable embryos (4.7 ± 2.9 vs. 3.7 ± 2.8, *p* = 0.002) was higher among cancer patients compared to age‐matched comparisons with male or tubal factor infertility. The total mean gonadotropin dose used was higher among cancer patients (2243 ± 963 IU vs. 1679 ± 765 IU, *p* < 0.001).

**Conclusion:**

We conclude that a good ovarian response during FP can be achieved in female cancer patients.

## Graphical abstract

A good ovarian response can be achieved in female cancer patients. Factors that affected the patient's decision to undergo fertility preservation included cancer type, gonadotoxicity and timeline of cancer treatment as well as motivation to have children.

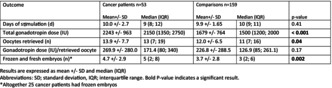

## 1 INTRODUCTION

Advances in multimodality cancer treatments have increased the long‐term survival rates for adolescents and young adults, now ranging from 83% to 86%.^1^, ^2^, ^3^ As survival rates increase, so does the risk for late complications. For female cancer survivors, these include impaired reproductive health and diminished reproductive function due to gonadotoxic cancer treatments, which could eventually lead to infertility.^4^ Fertility preservation (FP) programs have been developed and nowadays cryopreservation of oocytes, embryos or ovarian tissue are established technique, which can be used to optimize the fertility of cancer survivors.^5^, ^6^

According to recent recommendations,^5^, ^6^, ^7^ clinicians should, as early as possible, inform cancer patients about the impact that cancer treatment may have on their fertility. There are, however, limited published data on fertility counselling (FC), FP and FP cycle outcomes in female cancer patients with conflicting results. Two previous studies found that cancer patients had a poorer response to controlled ovarian hyperstimulation (COH),^8^, ^9^ whereas another found similar ovarian stimulation outcomes in breast cancer patients compared to patients undergoing elective FP.^10^ A systematic review^11^ comparing COH in cancer patients and healthy women between 1997 and 2015 found no significant differences in the number of oocytes retrieved. There are only limited data, consisting mainly of case studies,^12^, ^13^ on ovarian hyperstimulation syndrome (OHSS) in cancer patients, a serious, potentially lethal complication of COH that could delay the start of chemotherapy and worsen the prognosis of cancer.

The aim of our study was to identify factors that contribute to the decision to proceed to FP after FC in female cancer patients. In addition, we studied outcomes of FP in female cancer patients compared to a matched female comparison group. The primary outcome analysed was the number of oocytes retrieved and secondary outcomes included the number of stimulation days, total gonadotropin dose, gonadotropin dose/retrieved oocyte, number of transferrable embryos and cases of OHSS.

## 2 MATERIALS AND METHODS

In this register‐based cohort study, we used Helsinki University hospital records to identify all women, diagnosed with cancer at the age of 16–42 years, between 1 January 2011 and 31 December 2019, who received FC at the Reproductive Medicine Unit, Department of Obstetrics and Gynecology at Helsinki University Hospital. A combination of ICD‐10 diagnostic codes Z31.6 (General counselling and advice on procreation) and C* (malignancy) was used to identify female cancer patients receiving FC from the electronic records. The hospital district covers 2.2 million inhabitants (40% of the Finnish population) and is responsible for all public health care of FP in cancer patients. For every cancer patient undergoing FP, three female comparisons, matched by age at COH, time period, parity and smoking, with fertility treatments due to male factor (ICD‐10 diagnostic code N97.4) or tubal factor (ICD‐10 diagnostic code N97.1) infertility were identified. Detailed information on FC, FP and in vitro fertilization (IVF) treatments was received from the hospital records.

### 2.1 Fertility counselling and fertility preservation

In order to be included in the study, FC had to include all of the following; the patient had a visit at the clinic, a gynaecologist made an evaluation of the fertility of the patient, either by measurement of antral follicle count (AFC) and/or anti‐Müllerian hormone (AMH) serum level (μg/L), informed the patient about the possible impact of cancer treatment on fertility and informed the patient about possible FP techniques. In this study, FP was defined as cryopreservation of oocytes (CoO), embryos (CoE) or ovarian tissue (CoOT). OHSS was diagnosed using the OHSS classification by Golan,^14^ based on abdominal discomfort/pain, findings on ultrasound (ascites and enlarged ovaries) and laboratory findings.

A patient is entitled to FP in Finnish public health care if she meets the following criteria:

1. Age younger than 39 years (in CoOT the age limit is 35 years).
2. The cancer treatment is expected to be curative.
3. The patient does not have more than one previous child.
4. The FP is expected to be successful considering the potential risks (usually an AMH >0.2 ug/l and an AFC >5 is required).
5. The ovum pick‐up procedure can be performed safely.
6. A delay of 10–14 days (7 days for CoOT) before starting the cancer treatment is possible.
7. A hysterectomy has not been performed and is not planned (surrogacy is forbidden by the Finnish law)

FC, however, also includes information on contraception and how the cancer treatment might affect reproductive organs in general and will be given until the age of 43 years. The patients' wish to proceed to FP as well as reasons for declining it are always documented in the medical records during an FC. The gynaecologist' decision and reasons behind it are also documented in the medical records.

### 2.2 Study design

**FIGURE 1:**
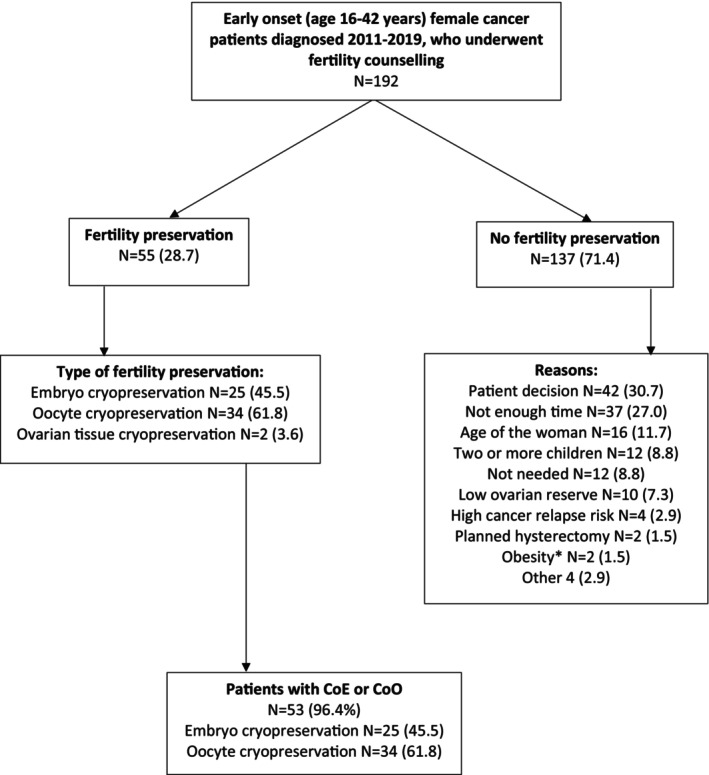
Results on fertility counselling in female cancer patients and fertility preservation methods used. * BMI 48 and 55. CoE, cryopreservation of embryos; CoO, cryopreservation of oocytes.

We included 192 female cancer patients, diagnosed between 1 January 2011 and 31 December 2019 at the age of 16–42 years, who received FC (Figure 1) in our data. Age at cancer diagnosis was categorized into seven groups (16–26, 27–28, 29–30, 31–32, 33–34, 35–36, 37–38 years) and calendar time at cancer diagnosis into three time periods (2011–2013, 2014–2016, 2017–2019). These age and calendar time groups were used to identify matched comparisons. Cancer types were categorized into breast cancer, Hodgkin's lymphoma, leukaemia, cancer in the digestive system, non‐Hodgkin's lymphoma, ovarian cancer, endometrial cancer, sarcomas and other cancers. When categorizing risk for infertility according to cancer treatment, we divided cancer patients into those with high (>80%), intermediate (20%–80%), low (<20%), very low or no risk and unknown risk for treatment‐related amenorrhea according to the ESHRE 2020 guidelines (ESHRE Female Fertility Preservation Guideline Development Group, 2020). Previous live births were divided into three groups (null, one or two or more).

Descriptive factors related to the fertility were: age at fertility treatment, AMH serum levels (μg/L), AFC at ultrasound and body mass index (BMI).

### 2.3 Statistical analysis

Results are presented as mean ± standard deviation (SD), as well as median and quartiles (25th and 75th quartiles). The normality of distributions was evaluated with the Kolmogorov–Smirnov test and the variables were found to be normally distributed. For descriptive data, the difference in categorical variables between cancer patients undergoing versus not undergoing FP was analyzed using the *χ* ^2^‐test. For the matching analysis, results were compared using Student t test. *p* values <0.05 were defined as significant. All analyses were conducted using STATA version 14.1 (StataCorp, College Station, TX, USA).

### 2.4 Ethics

This study protocol, including the use of medical record data without patient consent, was approved by the Research Committee of the Helsinki University Hospital. Separate approval from the Ethics Committee was not needed in this register‐based study, as no subjects or caregivers were contacted.

## 3 RESULTS

We identified 192 female cancer patients, diagnosed between 1 January 2011 and 31 December 2019 at the age of 16–42 years, who received FC (Figure 1). After careful evaluation by a reproductive endocrinologist, 97 (50.5%) cancer patients were eligible for FP. Of these, 55 (56.7%) underwent CoO, CoE or CoOT, whereas 42 (43.3%) declined. Reasons for not being eligible for FP included that there was not enough time before onset of cancer treatment (27.0%), age of the woman (11.7%), already having two or more children (8.8%), FP not needed (8.8%), low ovarian reserve (7.3%), high cancer relapse risk (2.9%), planned hysterectomy (1.5%) or obesity (1.5%). In our data, six cancer patients had both oocytes and embryos cryopreserved. Two patients underwent two FP cycles before cancer treatment. In these cases, we included the first FP cycle in our study. Altogether, 53 female cancer patients underwent CoO and/or CoE and two patients underwent CoOT. One cancer patient (1.9%) underwent an FP cycle with agonist protocol, the rest had an antagonist protocol. The benefit of an antagonist protocol is that it can be started at any time of the menstrual cycle, enabling a prompt start of the COH for cancer patients. Among female comparisons, 97 (61.0%) underwent a treatment cycle with agonist protocol. Since 2017, letrozole has been used as part of the antagonist protocol in breast cancer patients. In our data, 17 (51.5%) breast cancer patients used letrozole as part of their antagonist protocol. Among cancer patients, 9 (17%) used a trigger containing human chorionic gonadotropin (hCG), whereas 44 (83%) used an agonist trigger. Among comparisons, 156 (98.1%) used a trigger containing hCG, whereas 3 (1.9%) used an agonist trigger.

### 3.1 Outcomes of fertility counselling

The number of patients referred for FC increased significantly during the time period of our study and over half of the patients were diagnosed between 2017 and 2019 (Table 1). The most common cancer type among women attending FC was breast cancer (56.8%) and the second most common cancer was Hodgkin's lymphoma (23.4%). Almost half of all women attending FC (47.9%) were those with a high or intermediate risk for infertility. Most cancer patients with FC did not have previous children (83.3%). The proportion of cancer patients eligible and willing to undergo FP after FC did not change significantly over time and was 23.8% in 2011–2013, 32.8% in 2014–2016 and 25.5% in 2017–2019 (results not shown).

**TABLE 1:** Descriptive characteristics of female cancer patients who attended fertility counselling.

Characteristics	Cancer patients *n* (%) (*n* = 192)
Age at diagnosis (years)	
16–26	62 (32.3)
27–28	17 (8.9)
29–30	31 (16.2)
31–32	23 (12.0)
33–34	21 (10.9)
35–36	19 (9.9)
37–38	12 (6.3)
39–42	7 (3.7)
Year of diagnosis	
2011–2013	21 (10.9)
2014–2016	61 (31.8)
2017–2019	110 (57.3)
Cancer type	
Breast	109 (56.8)
Hodgkins'lymphoma	45 (23.4)
Leukemia	8 (4.2)
Digestive system	7 (3.7)
Non‐Hodgkins'lymphoma	6 (3.1)
Ovarian	6 (3.1)
Endometrial	2 (1.0)
Sarcomas	2 (1.0)
Others	7 (3.7)
Risk for infertility according to cancer treatment	
High risk (>80%)	20 (10.4)
Intermediate risk (20%–80%)	72 (37.5)
Low risk (<20%)	45 (23.4)
Very low or no risk	31 (16.2)
Unknown risk	17 (8.9)
Treatment not specified	7 (3.6)
Parity	
Null	160 (83.3)
One	24 (12.5)
Two or more	8 (4.2)

All in all, 137 (71.4%) women did not undergo FP after FC. The most common reason (*n* = 42, 30.7%) was that the patient did not want the procedure even though the gynaecologist and oncologist agreed that they met the criteria. For 37 women (27.0%), there was not enough time to perform a CoO and/or CoE before onset of cancer treatments (Figure 1).

Table 2 shows the characteristics of cancer patients undergoing FP and those declining FP despite being eligible for the procedure after FC. The most common cancer diagnoses among patients undergoing FP included breast cancer (*n* = 36, 65.5%) and Hodgkin's lymphoma (*n* = 9, 16.4%). Two cancer patients undergoing FP (3.6%) were considered to have a high risk for infertility, whereas most cancer patients with FP had an intermediate risk for infertility (22, 40.0%). There were no significant differences between age at cancer diagnosis, year of cancer diagnosis and parity but cancer patients undergoing FP had been recommended cancer treatments with a higher risk of infertility (*p* = 0.01) and they had more frequently breast cancer and less frequent lymphoma (*p* = 0.043) compared to women who declined FP (Table 2).

**TABLE 2:** Characteristics of cancer patients who underwent FP and cancer patients declining FP.

Characteristics	Cancer patients who underwent FP (*n* = 55)	Cancer patients who declined FP (*n* = 42)	*p*‐Value
Age at diagnosis (years)			
16–19	1 (1.82)	2 (4.76)	0.402
20–24	9 (16.36)	11 (26.19)	
25–29	17 (30.91)	14 (33.33)	
30–34	21 (38.18)	9 (21.43)	
35–39	7 (12.73)	6 (14.29)	
Year of diagnosis			
2011–2013	9 (16.36)	4 (9.52)	0.404
2014–2016	19 (34.54)	12 (28.57)	
2017–2019	27 (49.09)	26 (61.90)	
Risk for infertility according to cancer treatment			
High risk (>80%)	2 (3.64)	3 (7.14)	**0.011**
Intermediate risk (20%–80%)	22 (40.00)	9 (21.43)	
Low risk (<20%)	13 (23.64)	16 (38.10)	
Very low or no risk	8 (14.55)	13 (30.95)	
Unknown risk	10 (18.18)	1 (2.38)	
Cancer type			
Breast	36 (65.45)	21 (50.00)	**0.043**
Hodgkins'lymphoma	9 (16.36)	19 (45.24)	
Non‐Hodgkins'lymphoma	3 (5.45)	1 (2.38)	
Digestive system	2 (3.64)	0	
Ovarian	2 (3.64)	0	
Other	3 (5.45)	1 (2.38)	
Parity			
Null	54 (98.18)	39 (92.86)	0.191
One	1 (1.82)	3 (7.14)	

*Note*: Values are expressed as *n* (%).

Bold *p*‐value indicates a statistically significant.

Abbreviation: FP; fertility preservation.

The most common reason for a patient to decline FP, despite being eligible for the procedure, was the wish to focus on the upcoming cancer treatment and not to receive any additional medications or procedures during that (*n* = 27, 64.3%). Eight patients (19.0%) did not wish to delay the start of chemotherapy and seven (16.7%) felt that they did not wish to have children or biological children in the future (results not shown).

### 3.2 Cycle outcomes in women undergoing fertility preservation

The age range of cancer patients with CoO or CoE as FP was 19–38 years with the mean age at cycle start being 29.5 ± 4.7 years (Table 3). For cancer patients with FP, the mean AMH at cycle start was 4.3 ± 6.6 μg/L, the mean AFC was 17.9 ± 7.8 and the mean BMI was 23.8 ± 4.1, with no differences between cancer patients and their comparison group (Table 3). The mean total gonadotropin dose used among cancer patients was higher compared to female comparisons (2243 ± 963 IU vs. 1679 ± 764 IU, *p* < 0.001), as was the mean number of oocytes retrieved in female comparisons (13.9 ± 7.7 vs. 12.0 ± 6.5, *p* = 0.04) (Table 4). The mean number of transferrable embryos was also significantly higher among cancer patients compared to female comparisons (4.7 ± 2.9 vs. 3.7 ± 2.8, *p* = 0.002). Fresh embryo transfers were only performed in the comparison group (144, 90.6%). Mean number of stimulation days and gonadotropin dose per retrieved oocyte were similar in both groups (Table 4). Separate subgroup analyses comparing patients with breast cancer to women with other cancers revealed no differences in total gonadotropin dose used, oocytes or embryos retrieved or stimulation days (Table S1). We identified no cases of OHSS in the cancer patients undergoing FP, whereas among the comparisons we identified eight (5.0%) cases of mild OHSS.

**TABLE 3:** Characteristics of cancer patients undergoing fertility preservation cycles and their comparisons.

Characteristics	Cancer patients *n* = 53		Comparisons *n* = 159		*p*‐Value
	Mean ± SD	Median (IQR)	Mean ± SD	Median (IQR)	
Age at fertility treatment (years)	29.5 ± 4.7	30 (26; 33)	29.7 ± 4.2	30 (26; 33)	0.62
AMH (μg/L)	4.3 ± 6.6	3.5 (1.9; 4.4)	4.2 ± 3.1	3.2 (2; 5.6)	0.46
AFC (*n*)	17.9 ± 7.8	17.5 (13; 23)	18.8 ± 7.8	18 (12; 23)	0.74
BMI (kg/m^2^)	23.8 ± 4.1	22.7 (21; 25.7)	24.1 ± 4.0	23.2 (21; 26.5)	0.67

*Note*: Results are expressed as mean ± SD and median (IQR).

Abbreviations: AFC, antral follicle count; AMH, anti‐Müllerian hormone; BMI, body mass index; IQR, interquartile range.

**TABLE 4:** Comparison of ovarian stimulation cycles in cancer patients and their comparisons.

Outcome	Cancer patients *n* = 53		Comparisons *n* = 159		*p*‐Value
	Mean ± SD	Median (IQR)	Mean ± SD	Median (IQR)	
Days of stimulation (days)	10.0 ± 2.7	9 (8; 12)	9.9 ± 1.65	10 (9; 11)	0.41
Total gonadotropin dose (IU)	2243 ± 963	2150 (1350; 2750)	1679 ± 764	1500 (1200; 2000)	**<0.001**
Oocytes retrieved (*n*)	13.9 ± 7.7	13 (7; 19)	12.0 ± 6.5	11 (7; 16)	**0.04**
Gonadotropin dose (IU)/retrieved oocyte	269.9 ± 280.0	171.4 (80; 340)	226.8 ± 288.5	126.9 (85; 261.1)	0.17
Frozen and fresh embryos (*n*)^a^	4.7 ± 2.9	5 (2; 8)	3.7 ± 2.8	3 (2; 6)	**0.002**

*Note*: Results are expressed as mean ± SD and median (IQR). Bold *p*‐value indicates a significant result.

Abbreviation: IQR, interquartile range.

^a^ Altogether 25 cancer patients had frozen embryos.

## 4 DISCUSSION

In our study, 192 female cancer patients were referred for FC between 2011 and 2019. Even though half of these women fulfilled the criteria for FP, less than one‐third (55 women) underwent FP. Cancer patients undergoing FP had been recommended cancer treatments with a higher risk of infertility compared to women declining FP. Factors leading to cancer patients declining FP included wish to concentrate on the cancer treatment and recovery and to proceed with cancer treatment as soon as possible as well as a lack of desire to have children. Female cancer patients undergoing FP used significantly higher total doses of gonadotropin in their treatment cycles and the mean numbers of oocytes retrieved and embryos frozen were also higher compared to female comparisons.

In our study, cancer patients declining FP had, in general, been recommended a cancer treatment with lower risk for infertility compared to those undergoing FP. The risk estimation may thus have influenced the patient's decision‐making. Common reasons for declining FP among the patients in our data were the wish to proceed with cancer treatments as soon as possible and the lack of desire to have children. A previous study from 2008 found that cancer survivors are less motivated to have children because of the experienced cancer.^15^ However, a study from 2012 showed that even though cancer survivors are worried and unsure about their fertility, they were generally hopeful about becoming biological parent and also expressed positive feelings towards adoption and surrogacy.^16^ Two previous studies on patients´ decision‐making after FC^17^, ^18^ showed very conflicting results. A Swedish study^19^ conducted between 1998 and 2018, found that 73.2% of the women who were recommended FP decided to undergo the treatment. Notable is that this study included FC for malign, as well as benign indications (such as gynaecological conditions, autoimmune diseases or gender dysphoria), probably explaining the higher number of patients undergoing FP compared to our study. In another study on breast cancer patients,^18^ only 7% and 1% of all breast cancer patients with FC decided to undergo CoE and CoO respectively. In this study, there was, however, an increasing trend over time, as FP increased from 5% in 2006 to 15% in 2012. In our study, the proportion of cancer patients eligible and willing to undergo FP after FC was higher (24%–33%) and did not change significantly over time. Probably this is explained by FC being centralized to a few doctors and the indications for FP being the same during the study period.

The risk of infertility among cancer survivors is difficult to estimate, as it is variable and depends on several other factors than the gonadotoxic treatment, such as the age of the woman, ovarian reserve before cancer treatment and other comorbidities.^18^ Therefore, FC should be individualized and every patient should be considered as being at potential risk of developing treatment‐related infertility.^20^ Moreover, the risk of treatment‐related infertility still remains unknown for many chemoterapeutic agents and their combinations.^19^ Notable is also that the final pathologist report, based on which the cancer treatment is decided, is not always available when decision about FP has to be made. In our study, almost half of the women undergoing FP were estimated to have a high or intermediate risk for treatment‐related infertility. Still, for 18.1% of cancer patients undergoing FP, the risk of infertility was categorized as unknown, mirroring the challenge of estimating treatment‐related infertility with new cytotoxic drugs and treatment combinations.

Studies have suggested that AMH levels might be reduced at the time of diagnosis in some cancer types, including lymphomas and breast cancer patients with BRCA1 mutations.^21^, ^22^, ^23^ However, for breast cancer patients in general, AMH levels do not appear to be reduced.^23^, ^24^ In our study, cancer patients who underwent FP had similar AMH levels compared to comparisons. Cancer patients used significantly higher total doses of gonadotropin in their treatment cycles, but the mean numbers of retrieved oocytes and frozen embryos were also higher compared to female comparisons, resulting in a similar gonadotropin dose per retrieved oocyte in both groups. Notable is that the majority of comparisons had a fresh embryo transfer which was included in the total number of embryos in the analyses. When comparing breast cancer patients to other cancer patients in our data we found no significant differences in gonadotropin doses, oocytes or embryos retrieved.

A previous systematic review comparing COH in cancer patients and healthy women found no significant differences in gonadotropin doses, the number of oocytes retrieved or fertilization rates.^11^ Notable is that the women in our study had their fertility treatment between 2012 and 2019, whereas the women in the systematic review had their fertility treatments between 1997 and 2015. Possibly the introduction of GnRH trigger for final oocyte maturation, known to decrease the risk for hyperstimulation, and the increased use of letrozole during COH of breast cancer patients have decreased potential complication risks and led to fertility doctors feeling safer to use larger doses of gonadotropin, affecting our results. The antagonist protocol was commonly used among cancer patients because it enabled a flexible and prompt start of COH, irrespective of the phase of the menstrual cycle. Also, the risk of OHSS is significantly lower with antagonist protocol compared to agonist protocol. The agonist protocol was more often used among comparisons since ovum pick‐up is easier to schedule with this protocol. Systematic reviews^25^, ^26^ have, however, shown that the number of retrieved oocytes and embryos, as well as ongoing pregnancy rates were significantly lower in fertility treatments with antagonist protocol compared to agonist protocol in patients with normal ovarian function. Hence, the difference in stimulation protocols did not explain the higher number of oocytes and frozen embryos in cancer patients compared to female comparisons in our study.

The reason for a more aggressive gonadotropin stimulation in cancer patients compared to comparisons may be to optimize the oocyte yield for the single treatment cycle that is usually possible before the onset of cancer treatments. According to our results, cancer patients responded to the higher gonadotropin stimulation with a higher number of oocytes and embryos. Notable is that despite the higher gonadotropin doses and higher mean numbers of oocytes retrieved in cancer patients compared to comparisons in our study, there were no OHSS cases or bleeding complications after ovum pick‐up in cancer patients. Avoidance of OHSS and bleeding complications are important for cancer patients undergoing FP, as these complications could delay cancer treatments and increase the already higher risk for thromboembolism.^27^

The main strength of our study is outcome ascertainment by use of reliable, compulsory, hospital records from a single hospital, still covering 40% of the Finnish population, to obtain outcome data free of possible recall bias. Using hospital records enabled us to study important confounders for cancer patients and female comparisons, making it possible to adjust for different factors that might interfere with the outcomes. We were also able to categorize the risk of infertility based on detailed information on chemotherapeutical agents and doses, as well as radiotherapy. The main limitation of our study is that we lack information on all female cancer patients diagnosed in the hospital district of Helsinki and Uusimaa under the time period of our study and therefore do not know which factors lead to cancer patients being referred for FC. Probably these cancer patients consisted of a selected group regarding cancer treatment and reproductive history. For a majority of cancer patients undergoing FP, the procedure was done between 2017 and 2019, resulting in only a short follow‐up period, which is why we did not yet study the utilization rate of frozen oocyte and embryos or pregnancy rates after FP in our study. Another limitation is the relatively small number of women undergoing FP, which could affect our results. To increase the reliability of our results, we report both mean and median values for our outcomes. Although acknowledging this limitation, there is still limited recently published data on FC and FP cycle outcomes, and therefore, our results are valuable for clinicians treating young female cancer patients.

Over the last few years, increasing attention has been given to highlight the importance of FC for cancer patients. In our study, 28.6% of all cancer patients attending FC between 2011 and 2019 decided to undergo FP. Compared to previous results, our study mirrors a more active approach among clinicians towards offering FP for cancer patients during the more recent years. Similar results have also been observed regarding fertility treatments in cancer survivors. A Finnish study^28^ showed a significantly increased use of ART in cancer survivors from 2004 onwards.

## 5 CONCLUSION

We conclude that compared to previous results, our study mirrors a more active approach among clinicians towards offering FP for cancer patients during the more recent years. However, not all cancer patients eligible for FP are willing to undergo the procedure after receiving FC. Factors that might influence their decision are cancer type, gonadotoxicity of cancer treatment as well as the wish to concentrate on the cancer treatment and motivation to have children at that point. For female cancer patients deciding to undergo FP, a good ovarian response can be achieved. Long‐term follow‐up on later spontaneous pregnancies, as well as utilization rates of cryopreserved oocytes and embryos in cancer survivors, is needed in order to evaluate the long‐term need for FP in this patient group.

## AUTHOR CONTRIBUTIONS

**Johanna Melin:** Conceptualization (equal); data curation (equal); formal analysis (lead); funding acquisition (equal); investigation (equal); methodology (lead); project administration (equal); resources (equal); software (equal); supervision (equal); validation (equal); visualization (lead); writing – original draft (lead); writing – review and editing (equal). **Elina Holopainen:** Conceptualization (equal); data curation (equal); formal analysis (supporting); funding acquisition (equal); investigation (equal); methodology (supporting); project administration (equal); resources (equal); software (equal); supervision (equal); validation (equal); visualization (equal); writing – original draft (supporting); writing – review and editing (equal). **Hanna Savolainen‐Peltonen:** Conceptualization (equal); data curation (equal); formal analysis (supporting); funding acquisition (equal); investigation (equal); methodology (supporting); project administration (equal); resources (equal); software (equal); supervision (equal); validation (equal); visualization (equal); writing – original draft (supporting); writing – review and editing (equal).

## FUNDING INFORMATION

JM received funding from The Medical Society of Finland. HS‐P received funding from The Academy of Finland and The Finnish State Research Funding.

## CONFLICT OF INTEREST STATEMENT

EH reports a consulting honorarium from Merck and HS‐P has received lecture or consulting honoraria from Exeltis, Ferring, Merck and Orion. JM has no conflicts to declare.

## PRECIS

A good ovarian response during fertility preservation can be achieved in female cancer patients. Factors that affected the patient's decision to undergo fertility preservation included cancer type, gonadotoxicity and timeline of cancer treatment, as well as motivation to have children.

## Supporting information


Table S1.

## Data Availability

The data underlying this article cannot be shared publicly due to patient protection policies. Partial, fully anonymous data may be shared on reasonable request to the corresponding author.
