# Supplementary material for: Fertility counselling and fertility preservation among early onset female cancer patients—A Finnish register‐based study
Source: Cancer Med. 2024 Feb 24;13(3):e7034. doi: 10.1002/cam4.7034 (PMC10891448; doi:10.1002/cam4.7034)
Supplement: Supplementary file 1 — Table S1. [file CAM4-13-e7034-s001.docx]

**Supplementary table S1.** Comparison of ovarian stimulation cycles in patients with breast cancer to other cancers.

| Outcome | Breast cancer patients n=33  Mean+/- SD Median (IQR) | | Other cancers n=20  Mean+/- SD Median (IQR) | | p-value |
| --- | --- | --- | --- | --- | --- |
| Days of stimulation (d) | 10.3 +/- 3.0 | 10 (8; 12) | 9.35 +/- 2.06 | 9 (8; 10) | 0.90 |
| Total gonadotropin dose (IU) | 2295 +/- 964 | 2250 (1400; 2750) | 2156.25 +/- 980.0 | 2012.5 (1350; 2850) | 0.69 |
| Oocytes retrieved (n) | 14.2 +/- 7.6 | 12 (7; 20) | 13.45 +/- 7.9 | 13.5 (6.5; 16.5) | 0.63 |
| Gonadotropin dose (IU)/retrieved oocyte | 257.9 +/- 261.2 | 171.4 (88.0; 171.4) | 289.7 +/- 314.7 | 142.5 (77.5; 427.5) | 0.34 |
| Embryos frozen (n)* | 4.8 +/- 3.1 | 5 (2; 8) | 4.4 +/- 2.8 | 4 (2; 7) | 0.62 |

Results are expressed as mean +/- SD and median (IQR).

Abbreviations: SD; standard deviation, IQR; interquartile range.

*Altogether 15 breast cancer patients and 10 other cancer patients had frozen embryos
